# Demographic and Microbiological Profile of Corneal Ulcers in Eastern India

**DOI:** 10.7759/cureus.67259

**Published:** 2024-08-20

**Authors:** Neha Shilpy, Abhishek Chandra, Harshita Lal, Sarswati, Ritu Dixit, Govind V Khalkho, Diksha Sareen

**Affiliations:** 1 Ophthalmology, Netrodaya The Eye City LLP, Varanasi, IND; 2 Microbiology, HL Diagnostics, Varanasi, IND; 3 Genetics, Netrodaya The Eye City LLP, Varanasi, IND; 4 Ophthalmology, Prakash Netralaya, Varanasi, IND

**Keywords:** bacterial corneal ulcer, fungal corneal ulcer, antimicrobial susceptibility, microbiological testing, corneal ulcer

## Abstract

Purpose: To evaluate the microbiological profile of corneal ulcers presenting at a tertiary care eye hospital in eastern Uttar Pradesh (UP), India.

Methods: This retrospective, observational study included patients with corneal ulcers who underwent corneal scraping and microbiological examination of the sample from May 2014 to October 2023. The demographic details, predisposing factors, and clinical examination data of the patients were noted. Microbiology report of staining and culture (blood agar and Sabaraud’s dextrose agar) of corneal scraping sample was analyzed in percentage. Reports of antimicrobial susceptibility testing were also noted and analyzed.

Results: A total of 695 corneal scraping samples were examined during the study period. The mean age of the patients was 45.95 years. Among the patients, 412 (59.28%) were males and 283 (40.72%) were females. Among the patients, 402 (57.84%) belonged to an agricultural background. Trauma was the most common predisposing factor associated with 350 (50.36%) cases. The corneal scraping samples were stain-positive in 455 (65.47%) cases, of which, 130 (28.57%) were gram-positive and 325 (71.43%) were positive on potassium hydroxide (KOH) mount. Culture was positive in 306 (44.03%) cases, of which, bacterial isolates were found in 78 (25.49%), and fungal isolates were found in 228 (74.51%). *Fusarium* was the most common fungal isolate in 72 (31.58%) cases followed by *Aspergillus* in 60 (26.32%) cases. Among the bacterial isolates, *Staphylococcus aureus* was the most common in 20 (25.64%) cases followed by *Pseudomonas *and *Streptococcus*. The antimicrobial susceptibility testing showed that 47 (60.26%) of the bacterial isolates were sensitive to fluoroquinolones while the rest 31 (39.74%) were resistant. All the *Staphylococcus aureus* strains (including four cases of methicillin-resistant *Staphylococcus aureus*) were susceptible to vancomycin and linezolid, while 7 (35%) were resistant to moxifloxacin. None of the *Pseudomonas* strains were multidrug resistant. Among the fungal isolates, 220 (96.49%) were susceptible to voriconazole and 189 (82.89%) were sensitive to amphotericin B.

Conclusion: Fungal corneal ulcers are more common compared to bacterial ulcers in eastern India, particularly eastern UP and Bihar. This article highlights the importance of microbial testing and provides insight into the prevalent organisms and their antimicrobial susceptibility pattern in this geographic location, the knowledge of which will help clinicians in the appropriate management of these cases.

## Introduction

Corneal ulcers are a potentially blinding condition that accounts for a huge financial and social burden in developing countries [1,2]. It is a leading cause of corneal blindness in Asia, the Middle East, and Africa [1-2]. Predisposing factors include trauma, surgery, systemic diseases, use of topical corticosteroids, and contact lens usage [3]. The causative organisms can be bacteria, fungi, viruses, or protozoa [3].

Clinical diagnosis is often the basis for starting treatment in infectious corneal ulcers. However, the presentation is not always typical; so, broad-spectrum antibiotics and/or antifungals are prescribed on an empirical basis [4,5]. Due to the evidence of increasing resistance to commonly used antibiotics, and to prevent overuse, proper laboratory-confirmed diagnosis after corneal scraping is very important [4,5].

The microbiological profile varies with the geographic location and therefore it is important to know the current trends of causative organisms and their drug susceptibility [3]. Though there are studies in the literature that mention the prevalence of bacterial and fungal corneal ulcers in different parts of the world as well as in India, a study from eastern Uttar Pradesh (UP) and Bihar could not be found in the literature review. This study will highlight the current trends in etiological diagnosis and the anti-microbial susceptibility of corneal ulcer cases presenting at a tertiary care eye hospital in eastern UP.

## Materials and methods

This retrospective observational study was conducted at a tertiary care eye hospital in Varanasi, India. Data was obtained from the records for the period between May 2014 and October 2023. The study adhered to the tenets of the Declaration of Helsinki and written informed consent was taken from all the patients during sample collection regarding the possible use of data in research. As this was a retrospective observational study, therefore ethical clearance was waived off by the institutional review board.

We included patients who had been diagnosed with corneal ulcers and had undergone microbiological investigation of the corneal scraping sample collected at our hospital from May 2014 to October 2023. We excluded patients with viral keratitis and non-infective corneal ulcers. Demographic details, history of major predisposing factors, and clinical examination data of the patients were noted down from a register maintained for this purpose. Microbiology reports of the corneal scraping sample and reports of antimicrobial susceptibility testing were also noted.

As part of the standard protocol, corneal scraping was done in cases of infective corneal ulcers. After obtaining written informed consent, 0.5% proparacaine was instilled for topical anesthesia, and corneal scraping was done under a slit lamp. General anesthesia or sedation was required in children and uncooperative adults, where it was done under an operating microscope. Any mucus or debris on and around the ulcer was cleaned using a sterile swab stick. Then with a surgical blade no 15, the leading edges and base of the ulcer were scraped and directly inoculated into culture plates (blood agar and Sabouraud’s dextrose agar) for culture and sensitivity testing. Smears were also prepared by gently transferring the material onto two glass slides for gram staining and potassium hydroxide (KOH) wet mount preparation.

Bacterial isolates were identified as lactose fermenting (LF) (pink colonies) and non-lactose fermenting (NLF) (translucent colonies resembling the color of media) as per their growth on MacConkey agar. Oxidase test was performed on NLF colonies and further speciation of LF and NLF oxidase negative colonies was done by inoculating pure colonies on biochemicals (triple sugar iron, sulfur indole motility media, sugar fermentation media, Simon's citrate test, urease hydrolysis test) and interpreted after 18-24 hours of inoculation. Gram-positive colonies from blood agar were further identified by gram staining, catalase, and coagulase tests. Yeast-like colonies were identified by gram staining and filamentous fungi were identified by lacto-phenol cotton blue staining of fungal hyphae.

Antimicrobial susceptibility testing was also done as a routine procedure using the Kirby-Baeur disc diffusion method. At first, a broth of the organism grown (bacteria/fungi) was made in saline, the concentration depending on the specific organism. Then it was swabbed on a Mueller-Hinton agar plate and the required discs of antimicrobial drugs were applied noting the distance between the disc in consideration and the incubated growth [6]. The interpretation was done as per the Clinical and Laboratory Standards Institute guidelines [7]. The choice of antibiotics for susceptibility testing was based on the specific organism [8].

The demographic details and the microbiology results of the patients were entered in Microsoft Excel (Microsoft Corporation, Redmond, WA) and the data was analyzed. The percentage of culture-positive cases with the proportion of bacterial and fungal isolates and their anti-microbial susceptibility was calculated.

## Results

Data from a total of 695 patients with corneal ulcers were taken for the study (Table 1). The mean age of the patients was 45.95 years. Among the patients, 412 (59.28%) were males and 283 (40.72%) were females. Among the patients, 402 (57.84%) were farmers by occupation. Table 1 shows the occupation of the patients included. Trauma was the most common predisposing factor found in 350 (50.36%) patients, followed by self-medication/steroid use in 272 (39.14%) patients, systemic illness in 71 (10.22%) patients, and immunosuppressive treatment in two (0.29%) patients. 

**Table 1:** Occupation of the patients diagnosed with infective corneal ulcer

Occupation	Number of patients (%)
Farmer	402 (57.84)
Labourer	198 (28.49)
Student	49 (7.05)
Housewife	46 (6.62)
Total	695

The corneal scraping samples were stain positive in 455 (65.47%) cases, of which, 325 (71.43%) were KOH positive and 130 (28.57%) were detectable on gram stain with 85 (65.38%) being gram-positive, and 45 (34.62%) being gram-negative. Culture was positive in 306 (44.03%) cases, of which, bacterial isolates were found in 78 (25.49%), and fungal isolates were found in 228 (74.51%). The most common bacterial species isolated was *Staphylococcus aureus* followed by *Pseudomonas *and *Streptococcus*. The most common fungal species isolated was *Fusarium *spp. followed by *Aspergillus *spp. Table 2 shows the bacterial species isolated and Table 3 shows the fungal species isolated.

**Table 2:** Bacterial species isolated from corneal scraping samples in patients with corneal ulcer

Bacterial species isolated	Number of cases (%)
Staphylococcus aureus	16 (20.51)
Methicillin-resistant Staphylococcus aureus	4 (5.13)
Streptococcus	14 (17.95)
Pseudomonas	16 (20.51)
Klebsiella	1 (1.28)
Corynebacterium diphtheriae	1 (1.28)
Micrococci	1 (1.28)
Chryseobacterium indolegenes	2 (2.56)
Enterococcus faecium	4 (5.13)
Citrobacter freundii	4 (5.13)
Nocardia	3(3.85)
Unidentified bacteria	12 (15.38)
Total	78

**Table 3:** Fungal species isolated from corneal scraping samples in patients with corneal ulcer

Fungal species isolated	Number of cases (%)
Aspergillus	60 (26.32)
Fusarium	72 (31.58)
Candida	8 (3.51)
Mucor	6 (2.63)
Rhizopus	4 (1.75)
Bipolaris	7 (3.07)
Curvularia	6 (2.63)
Alternaria	2 (0.88)
Coletotrichum	2 (0.88)
Acremonium	1 (0.44)
Cladophialophora	5 (2.19)
Cladosporium	1 (0.44)
Hyalohyphomycosis	1 (0.44)
Scedosporium	1 (0.44)
Syncephalastrum	1 (0.44)
Unidentified fungi	51 (22.37)
Total	228

The results of antibiotic susceptibility testing are shown in Table 4 and Table 5 for the bacterial isolates and fungal isolates respectively. The antimicrobial susceptibility testing showed that 47 (60.26%) of the bacterial isolates were sensitive to fluoroquinolones while the rest 31 (39.74%) were resistant. There were four (20%) cases of methicillin-resistant *Staphylococcus aureus* (MRSA). All the *Staphylococcus aureus* strains (including MRSA) were susceptible to vancomycin and linezolid, while seven (35%) were resistant to moxifloxacin. None of the *Pseudomonas* strains were multidrug resistant. Among the fungal isolates, 220 (96.49%) were susceptible to voriconazole and 189 (82.89%) of the fungal isolates were sensitive to amphotericin B.

**Table 4:** Antibiotic susceptibility of isolated bacterial species in corneal ulcer scraping sample * antibiotic susceptibility not done for all bacterial species, and therefore percentage not mentioned - sensitivity not tested against this antibiotic

Bacterial species isolated	Total number (%) of organisms	Number (%) of organisms susceptible to cephalosporins	Number (%) of organisms susceptible to fluoroquinolones	Number (%) of organisms susceptible to aminoglycoside	Number (%) of organisms susceptible to vancomycin	Number (%) of organisms susceptible to linezolid	Number (%) of organisms susceptible to polymixin	Number (%) of organisms susceptible to ,eropenem	Number (%) of organisms susceptible to piperacillin + tazobactam
Staphylococcus aureus	16 (20.51)	-	12 (75.0)	-	16 (100)	16 (100)	-	-	-
Methicillin-resistant Staphylococcus aureus	4 (5.13)	-	1 (25.0)	3 (75)	4 (100)	4 (100)	-	-	-
Streptococcus	14 (17.95)	-	13 (92.86)	12 (85.71)	14 (100)	14 (100)	-	-	-
Pseudomonas	16 (20.51)	14 (87.5)	7 (43.75)	0	-	-	-	16 (100)	16 (100)
Klebsiella	1 (1.28)	-	1 (100)	1 (100)	-	-	1 (100)	1 (100)	-
Diphtheria	1 (1.28)	-	0	1 (100)	-	-	-	-	-
Micrococci	1 (1.28)	-	0	1 (100)	1 (100)	-	-	-	-
Chryseobacterium indolegenes	2 (2.56)	-	2 (100)	-	2 (100)	-	-	-	-
Enterococcus faecium	4 (5.13)	-	1 (25)	1 (25)	2 (50)	4 (100)	-	-	-
Citrobacter freundii	4 (5.13)	4 (100)	0	3 (75)	-	-	-	-	4 (100)
Nocardia	3(3.85)	-	1 (33.33)	3 (100)	-	-	-	-	-
Unidentified	12 (15.38)	12 (100)	9 (75)	12 (100)	-	-	-	-	-
Total	78	30^*^	47 (60.26)	37 ^*^	39^*^	38^*^	1^*^	17^*^	20^*^

**Table 5:** Antifungal susceptibility of isolated fungal species in corneal ulcer scraping sample

Fungal species isolated	Total number of organisms	Number (%) of organisms susceptible to amphotericin B	Number (%) of organisms susceptible to voriconazole	Number (%) of organisms susceptible to itraconazole	Number (%) of organisms susceptible to fluconazole
Aspergillus	60 (26.32)	47 (78.33)	60 (100)	48 (80)	50 (83.33)
Fusarium	72 (31.58)	53 (73.61)	72 (100)	11 (15.28)	54 (75)
Candida	8 (3.51)	8 (100)	8 (100)	7 (87.5)	7 (87.5)
Mucor	6 (2.63)	6 (100)	0	0	0
Rhizopus	4 (1.75)	4 (100)	3 (75)	1 (25)	1 (25)
Bipolaris	7 (3.07)	7 (100)	7 (100)	6 (85.71)	4 (57.14)
Curvularia	6 (2.63)	6 (100)	6 (100)	2 (33.33)	5 (83.33)
Alternaria	2 (0.88)	2 (100)	2 (100)	0	1 (50)
Coletotrichum	2 (0.88)	2(100)	2 (100)	2 (100)	2 (100)
Acremonium	1 (0.44)	0	1 (100)	0	0
Cladophialophora	5 (2.19)	5 (100)	5 (100)	5 (100)	4 (80)
Cladosporium	1 (0.44)	1 (100)	1 (100)	1 (100)	1 (100)
Hyalohyphomycosis	1 (0.44)	0	1 (100)	0	0
Scedosporium	1 (0.44)	0	1 (100)	0	0
Syncephalastrum	1 (0.44)	1 (100)	0	0	0
Unidentified	51 (22.37)	47 (92.16)	51 (100)	24 (47.06)	32 (62.75)
Total	228	189 (82.89)	220 (96.49)	83 (36.40)	129 (56.58)

## Discussion

Corneal ulcers are one of the leading causes of preventable corneal blindness [2]. Complications and sequelae of corneal ulcers, which include perforation, scarring, and anterior staphyloma, are vision-threatening. Therefore, proper and timely management of ulcer cases guided by knowledge of causative agents plays a crucial role in the prognosis of these cases.

The mean age of the patients in our study was 45.95 years. A review article on infectious keratitis by Ting et al. has mentioned that in most studies the age group between 30 to 55 years is seen to be mostly affected as that is the working age group [9]. The etiology of corneal ulcers varies between geographical locations, depending on the climatic conditions and the demographic profile of the population [10]. In our geographic location, a large part of the population thrives as agricultural workers or manual laborers. This population is quite susceptible to trauma and hence in our study, we found that male sex (59.28%), agricultural background (57.84%), and trauma (50.36%) were the most common predisposing factors. The review article by Ting et al. mentions male preponderance with trauma as the most common predisposing factor in studies from Asia, Africa, and South America similar to our study [9]. However, studies from the developed world like those from the UK and North America found females to be more commonly affected [11,12]. The reason for this was contact lens wear which was more prevalent in females and was the major predisposing factor in these studies. A study by Pramanick et al. from eastern India has found an even greater male preponderance (72.5%) which may be due to a smaller sample size [8].

In the present study, 44.03% of cases were culture-positive, and out of them, 74.51% had fungal growth while 25.49% had bacterial growth. While 130 cases were detectable on gram stain, only 78 showed bacterial growth. Similarly, out of the 325 KOH-positive cases, fungal isolates were found in 228 of them. This can be explained due to previous treatment with antibiotics and antifungals in many cases before presentation at our center, which hinders the growth of the organisms. Also, the other organisms might be fastidious or the culture and incubation settings were not suitable for their growth.

Ting et al. have mentioned in their review article that while bacteria are the most common causative organisms in infectious keratitis cases in most regions, fungal keratitis is more common in Asia [9]. Bacterial keratitis accounts for a majority in regions including Europe (91-93%) [11,13], North America (86-92%) [14], South America (79-88%) [15,16], Middle East (91.8%) [17], and Australia (93-100%) [18,19]. Contrary to this, in the Asian continent, particularly in India and China, fungi have been the predominant cause of infectious keratitis cases [20-22]. The predominance of fungi in India can be explained by the tropical climate and the majority of the agricultural population which predisposes them to trauma by vegetative matter.

In the present study, *Fusarium* spp. was the most common fungal isolate (31.58%) followed by *Aspergillus *spp. (26.32%). Other studies from India and China have also reported *Fusarium* (13-24%) and *Aspergillus *(8-30%) as the commonest fungi [20-22]. *Fusarium* was found to be the predominant fungi in China (26%) and India (31%) in a large study published in 2002 including eight Asian countries [2,23]. Filamentous fungi like *Aspergillus *and *Fusarium *thrive well in tropical climates while yeast-like fungi such as *Candida *thrive well in the temperate climates of the UK, Europe, and North America [24].

Among the bacterial isolates, *Staphylococcus aureus* was the most common (25.64%) followed by *Pseudomonas *spp. and *Streptococcus* spp. Other studies from India also have similar findings. Singh et al., in their study published in 2020, have found *Staphylococcus* to be the most common causative organism (47.1%) in pediatric infectious keratitis cases in North India [25]. Pramanick et al. have also found *Staphylococcus *aureus to be the most common followed by *Streptococcus *in eastern India in their study published in 2022 [8]. Another study from South India by Ranjini and Waddepally, published in 2016, has also found *Staphylococcus aureus *as the most common bacterial isolate followed by *Pseudomonas *and *Streptococcus *[26]. However, studies from other continents mention coagulase-negative *Staphylococcus* (CoNS) as the most common bacterial isolate followed by *Staphylococcus aureus*, *Pseudomonas*, and *Streptococcus *[11,13-19]. This difference can be due to the major predisposition to trauma in our country compared to other continents and also due to geographic variation. CoNS are a common ocular commensal and can cause infection whenever corneal epithelial integrity is disrupted while other organisms come from external sources during trauma.

The antimicrobial susceptibility testing revealed that 220 (96.49%) of the fungal isolates were susceptible to voriconazole, thereby proving it to be a very effective antifungal drug in these cases. Amphotericin B was also quite effective with 189 (82.89%) of the fungal isolates being sensitive. Among the bacterial isolates, fluoroquinolone resistance was seen in 31 (39.74%) samples. All the *Staphylococcus aureus* strains (including MRSA) were susceptible to vancomycin and linezolid, while 7 (35%) were resistant to moxifloxacin. Chang et al. and Galvis et al. have also mentioned vancomycin and linezolid as very effective treatment options for gram-positive infections, particularly *Staphylococcus* isolates [27,28]. Aminoglycosides, meropenam, and piperacillin along with tazobactam work well against gram-negative infections. None of the *Pseudomonas *strains we found were multidrug resistant.

The limitations of the current study are that antifungal sensitivity against natamycin (the most commonly used topical antifungal) could not be tested as natamycin disc is not available in India. Also, the clinical response based on antimicrobial susceptibility was not assessed as part of this study and we can conduct future studies on that.

## Conclusions

Male gender, trauma, and agricultural background are the most common predisposing factors for corneal ulcers in eastern India, particularly eastern Uttar Pradesh and Bihar. Fungal corneal ulcers are more common as compared to bacterial ulcers in our geographical setup. *Fusarium *is the most common fungal species and *Staphylococcus aureus *is the most common bacterial species causing corneal ulcers.

This article highlights the importance of microbial testing and provides insight into the prevalent organisms and their antimicrobial susceptibility pattern in our geographic location. This knowledge of trends will help clinicians in the appropriate management of these cases.
